# Autotransporter-Mediated Display of Complement Receptor Ligands by Gram-Negative Bacteria Increases Antibody Responses and Limits Disease Severity

**DOI:** 10.3390/pathogens9050375

**Published:** 2020-05-14

**Authors:** Kristen M Holland-Tummillo, Lauren E Shoudy, Donald Steiner, Sudeep Kumar, Sarah J Rosa, Prachi Namjoshi, Anju Singh, Timothy J Sellati, Edmund J Gosselin, Karsten RO Hazlett

**Affiliations:** 1 Department of Immunology and Microbial Disease, Albany Medical College, Albany, NY 12208, USA; Tummilk@amc.edu (K.M.H.-T.); SteineD@amc.edu (D.S.); KumarS@amc.edu (S.K.); SarahJRosa.82@gmail.com (S.J.R.); namjoshiprachi6@gmail.com (P.N.); GosselE@amc.edu (E.J.G.); 2 Department of Regenerative and Cancer Cell Biology, Albany Medical College, Albany, NY 12208, USA; CowenL@amc.edu; 3 Department of Infectious Disease, Southern Research Institute, Birmingham, AL 35211, USA; anjusing@uab.edu (A.S.); Timothy.Sellati@globallymealliance.org (T.J.S.)

**Keywords:** plug & play, vaccine-targeting, gram-negative, complement, autotransporter, tularemia

## Abstract

The targeting of immunogens/vaccines to specific immune cells is a promising approach for amplifying immune responses in the absence of exogenous adjuvants. However, the targeting approaches reported thus far require novel, labor-intensive reagents for each vaccine and have primarily been shown as proof-of-concept with isolated proteins and/or inactivated bacteria. We have engineered a plasmid-based, complement receptor-targeting platform that is readily applicable to live forms of multiple gram-negative bacteria, including, but not limited to, *Escherichia coli*, *Klebsiella pneumoniae*, and *Francisella tularensis*. Using *F. tularensis* as a model, we find that targeted bacteria show increased binding and uptake by macrophages, which coincides with increased p38 and p65 phosphorylation. Mice vaccinated with targeted bacteria produce higher titers of specific antibody that recognizes a greater diversity of bacterial antigens. Following challenge with homologous or heterologous isolates, these mice exhibited less weight loss and/or accelerated weight recovery as compared to counterparts vaccinated with non-targeted immunogens. Collectively, these findings provide proof-of-concept for plasmid-based, complement receptor-targeting of live gram-negative bacteria.

## 1. Introduction

From the discovery of a new pathogen, it can be many years before a vaccine that is specific to the organism is developed. Current methods involve the identification of virulence factors and pathogen-specific antigens (Ags) to compose subunit vaccines. Alternatively, inactivated whole cell vaccines are often non-stimulatory and require the addition of adjuvant for production of an effective immune response [1]. While adjuvants are effective at increasing innate immune activation, several drawbacks (non-specific immune cell activation, potential side effects, such as Guillain-Barré syndrome and myofasciitis, limited selection of approved adjuvants for the mucosal route) have prompted the search for adjuvant-free methods of vaccination [2]. Mucosal administration has become an appealing needle-free method of vaccination and several labs are developing new immunostimulatory adjuvants for this route, some of which are reviewed here [3,4]. Further, efforts to increase specificity and amplitude of the immune response to an Ag include targeting vaccines or other therapies to specific cells. For example, dendritic cells (DCs) present Ag via MHCI and MHCII, inducing both the cellular and humoral arms of adaptive immunity, which is an appealing feature in vaccine development [5,6]. Targeting receptors on DCs via the fusion of peptide Ags to either α-Clec9A antibody (Ab) [7], α-CD11c Ab, or DC-specific cytokines, results in increased Ag-specific Ab production [8], and/or increased T cell proliferation in vaccinated mice [9,10,11]. It is worth noting that, when tested side-by-side, these targeted Ags were more effective vaccines than their respective non-targeted Ag plus adjuvant. Besides fusion proteins, nanoparticles have also been engineered to interact with specific targets, such as mannose receptor on APCs for the delivery of DNA vaccines [12] or cancer cell-targeted particles for the delivery of chemotherapy agents [13]. This concept of activating specific immune cells against specific Ags, as opposed to broad treatments, has greatly advanced the treatment of cancer patients through directed drug delivery and immunotherapy [5,14,15,16].

We have shown that targeting whole bacteria to receptors on immune cells enhances vaccine efficacy. Specifically, when a monoclonal antibody (mAb) that was specific for the LPS of *Francisella tularensis* (*Ft*) was added to inactivated *Ft* (i*Ft*), the resulting mAb-i*Ft* Live Vaccine Strain (LVS) immunogen was targeted to Fc receptors (FcRs). Targeting Ag to FcRs in this way enhanced the processing and presentation of the Ag and conferred immunity to challenge against *Ft* LVS and human-virulent *Ft* SchuS4 [17,18]. While this approach was effective, it depended on having a pre-existing mAb―a situation that likely would not be the case for an emerging pathogen. Accordingly, we sought to develop a broadly applicable targeting approach that would eliminate this potential problem. Our goal was to develop a self-replicating plasmid that could be transformed into a bacterium, resulting in a targeted bacterium for use as a vaccine immunogen. A central advantage of this plasmid-based approach is that generating an infinite supply of targeted vaccine only requires an initial transformation event (such as electroporation) of the bacterium, and the ability to grow the transformed bacteria—capabilities of most public health agencies even in resource-challenged settings. Conceptually, it would have been desirable to clone the Fc domain of IgG as a fusion to a bacterial outer membrane protein (OMP), thereby mimicking the FcR-targeting effect of surface-bound Ab. However, we considered the technical challenges of expressing a functional, disulfide-linked and glycosylated Fc domain on the surface(s) of multiple gram-negative bacteria (without specialized modification of each bacterium) to be prohibitive. Accordingly, we considered alternative ligand-receptor pairings that would be more amenable to our goals and pursued the C3-complement receptor (CR) interaction.

During activation of the mammalian complement cascade, C3 is proteolytically cleaved and, one of the fragments, C3b, becomes covalently linked to the surface of susceptible microorganisms [19,20,21]. Further proteolytic activity of the bound C3b moiety first yields iC3b, and finally the terminal degradation product, C3d. Pathogen-bound iC3b and C3d are ligands for CRs primarily found on immune cells. C3d engendered significant interest as a “molecular adjuvant” following the demonstration that Ags linked to C3d multimers were more potent inducers of Ab than the Ags alone [22]. In fact, C3d multimers were observed to be as potent as complete Freund’s adjuvant. In this and latter studies, the adjuvant-like properties of C3d were determined to be partially, although not exclusively, CR2-dependent [23], raising the possibility that C3d might have mechanisms beyond binding CR2 on B cells and follicular DCs (FDCs) to stimulate immunity and establish immunological memory. Notably, recent findings indicate that C3d can also be bound by CR3—either alone or in a stable three-way complex of CR2-C3d-CR3 [19,24]. While the implications of C3d–CR3 interaction are likely yet-to-be fully defined, it is postulated that this three-way interaction might facilitate the trafficking of complement-opsonized Ags within lymph nodes between CR3+ macrophages (MΦs) and CR2+ B cells and/or FDCs [24]. Central to our choice of C3d as a ligand for our genetic-targeting approach were several reports of functional recombinant C3d-fusion proteins (expressed in *Escherichia coli* [*Ec*]), which, following purification, bound CR2 on B cells and/or enhanced Ab responses in vaccinated animals [25,26,27,28,29]. These data (published prior to the reports of C3d-CR3 binding) suggested to us that other bacteria (including those considered serum/complement resistant, such as *Ft* and *Klebsiella pneumoniae* [*Kp*]) might also be able to produce functional C3d—possibly as a fusion to an OMP. Moreover, reports that a minimal CR2-binding domain of C3d resided within a linear 28 residue fragment (p28) [21] suggested to us that p28 might be similarly amenable to bacterial surface display. Indeed, others have linked parasite-derived peptides to p28-OMP fusions for delivery by attenuated *Salmonella* and noted that these live vaccines induced elevated parasite-specific Ab responses [30].

**Figure 1:**
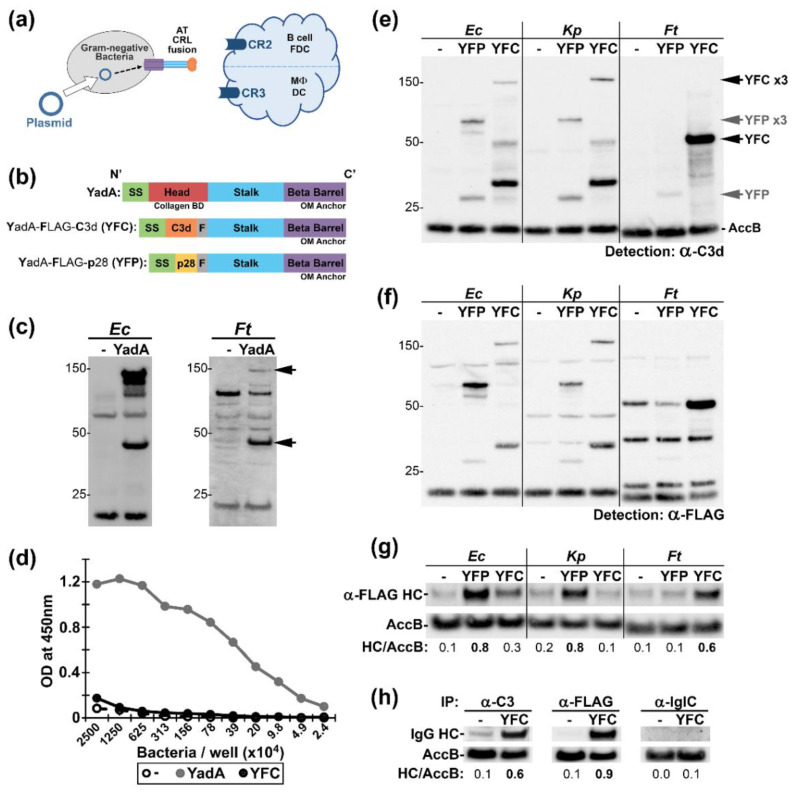
Conception and applicability of autotransporter (AT)—complement receptor ligand (CRL) fusions. (**a**) Graphical abstract. (**b**) The modification of YadA to yield YFC (YadA-FLAG-C3d) and YFP (YadA-FLAG-p28); SS: signal sequence. F: FLAG tag. (**c**) Heterologous expression of plasmid-borne YadA by *Ec* and *Ft* detected by western blot with α-YadA sera followed by horseradish peroxidase (HRP)-conjugated secondary Ab. Strains bearing empty vector are denoted by “-”. Arrows indicate the trimers and monomers of unmodified YadA. (**d**) Collagen-coated ELISA plates were incubated with serial dilutions of intact *Ft*:-, *Ft*:YadA, and *Ft*:YFC; bound bacteria were detected by α-*Ft* LPS Ab. (**e**,**f**) Whole cell lysates of *Ec*, *Kp*, and *Ft* containing empty vector (-) or the YFP or YFC expression vectors were probed by western blot with primary Ab specific for C3d and the FLAG epitope followed sequentially by biotinylated secondary Abs and streptavidin-HRP (SA-HRP). YFC and YFP trimers and monomers are designated with black and grey arrows. The ~20 kDa bands evident in all lanes are endogenously biotinylated bacterial proteins (annotated AccB in *Ec* and *Ft* [40]) detected by SA-HRP. (**g**) Intact bacteria as indicated were incubated in solution with α-FLAG Ab. (**h**) Intact *Ft* as indicated were incubated in solution with the indicated Ab. IglC is primarily a cytoplasmic *Ft* protein. Washed bacteria in (**g**,**h**) were probed for IgG heavy chain (HC), followed by biotinylated secondary Abs and SA-HRP.

Here our aim was to engineer a broadly applicable CR-targeting platform. We used the C-terminal β-barrel OM-insertion domains of an autotransporter (AT) to display C3d or p28 on bacterial surfaces. ATs are a class of gram-negative OMPs whose transport and insertion into the OM is widely-conserved, largely self-directed, and results in surface exposure of the passenger domain [31,32]—here engineered to be a CR-ligand. The relative independence of these proteins lends utility to their use in multiple gram-negative pathogens. We have linked C3d (or p28), along with a FLAG tag, to the N-termini of the β-barrel OM domain from a well-characterized AT, the trimeric *Yersinia enterolitica* adhesion protein A (YadA). Our rationale for the trimer was that clusters of surface-C3d should increase the avidity for CRs and potentially cross-link these receptors to enhance responses. We assessed expression and surface accessibility of our engineered proteins in three gram-negative bacteria: *Ec*, *Kp*, and *Ft*. We hypothesize that the CR-targeted bacteria, administered as adjuvant-free vaccines, would interact with CR-expressing cells (Figure 1a) and elicit greater specific Ab responses. Using *Ft* as a model, we have further characterized our novel whole-cell-targeting approach and observed increased association with and signaling in MΦs. Excitingly, mice that were vaccinated with the CR-targeted strain also produced elevated specific Ab and were protected from weight loss following subsequent *Ft* challenge. 

## 2. Results

### 2.1. Conception and Applicability of Autotransporter (AT)—Complement Receptor Ligand (CRL) Fusions to Multiple Gram-Negative Bacteria

Previously, we used an opsonizing Ab that was specific for the surface of *Ft* (mAb α-Ft LPS, IgG2a) to target *Ft* LVS vaccines to FcRs [17,18]. While this approach enhanced vaccine efficacy, it relied upon having pre-existing pathogen-specific reagents. Here, we sought to develop a rapid and broadly-applicable method of targeting intact bacteria to immune cells and pursued bacterial surface-expression of CR ligands (CRLs) (Figure 1a). We constructed our targeting platform on the backbone of the *Yersinia enterocolitica* (*Ye*) OMP YadA (Yersinia Adhesion A). YadA is an autotransporter with a N-terminal signal sequence, a collagen-binding domain (CBD), helical stalk, and a C-terminal β-barrel that anchors the protein into the outer membrane (OM) (Figure 1b). In *Ye*, mature YadA is a heat-resistant 132 kDa trimer, in which the stalk and CBD are surface-exposed [33,34,35]. We expressed YadA on a broad-host range plasmid in *Ec* and *Ft* and observed by western blot ~44 kDa and ~132 kDa α-YadA reactive bands consistent with the detection of monomers and trimers (Figure 1c). In *Ec*, ~50% of YadA appeared to be trimeric, while, in *Ft*, the trimeric form accounted for ~15% of YadA (not shown). In *Ft*, α-YadA reactive bands partitioned into the sarkosyl-insoluble phase consistent with that of an OMP (Figure S1a) and YadA-expressing *Ft* displayed robust collagen-binding (Figure 1d). 

We next replaced the collagen-binding domain in YadA with a FLAG tag and the murine complement protein C3d (a ligand for both CR2 and CR3 [24,36,37]) and termed this fusion YFC (YadA-FLAG-C3d). Similar to unmodified YadA, YFC partitioned as expected for an OMP (Figure S1b,c). We also engineered a fusion harboring the minimal CR2-binding fragment of C3d, p28 [38], in order to yield YadA-FLAG-p28 (YFP) (Figure 1b). We assessed YFC and YFP in three different γ-proteobacteria: *Ec*, *Kp*, and *Ft*. Lysates of recombinant bacteria were probed with α-C3d (Figure 1e) or α-FLAG (Figure 1f). The expression profiles of the AT-CRLs within *Ec* and *Kp* are similar with both monomers and trimers apparent. In the more distally related *Ft* i) YFC was more readily detected than YFP and ii) YFC monomers were more apparent than trimers—similar to the case with unmodified YadA. With enhanced detection, YFC-trimers are apparent in *Ft*, albeit at lower levels than in *Ec* (Figure S2). Together, these results suggest that YadA-CRL fusions and heterologously-expressed YadA trimerize to a roughly similar extent within a given bacterium. Next, we tested the surface-accessibility of our fusions by using intact bacteria to immunoprecipitate (IP) Ab specific for FLAG, C3, or the cytoplasmic *Ft* protein, IglC [39], with the latter serving as a negative control (Figure 1h). YFP supported robust Ab IP by *Ec* and *Kp*, whereas YFC appeared similarly effective in *Ft* (Figure 1g). These observations suggested that *Ec*:YFP, *Kp*:YFP, or *Ft*:YFC could be tested as vaccines in models of *Ec*, *Kp*, or *Ft* infection, respectively. As C3d interacts with two immune receptors (CR2 and CR3), we reasoned that analysis of YFC provided the best opportunity to detect the impact of CR-targeting in vivo. Accordingly, we pursued analysis of *Ft*:YFC and tularemia models of infection.

### 2.2. Bacterial Expression of YFC Enhances Binding and Uptake by Murine Macrophages

**Figure 2:**
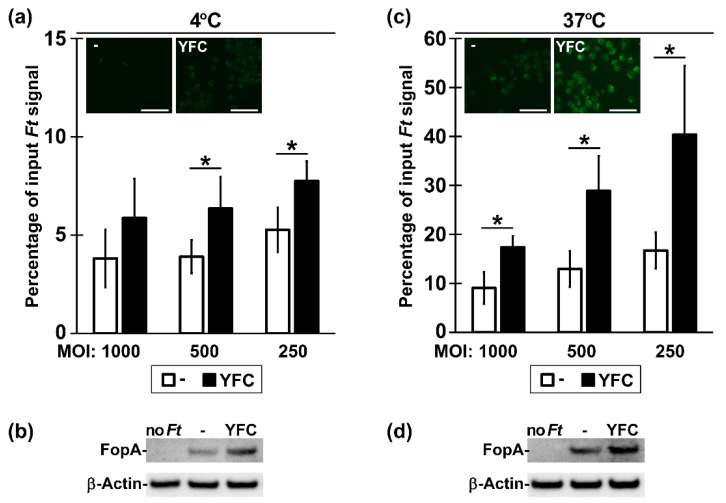
Bacterial expression of YFC enhances binding and uptake by MΦs. SYTO-stained *Ft* strains (- and YFC) were incubated at various MOIs with RAW cells for 2 h at 4 °C to assess binding (**a**) or at 37 °C to allow for bacterial uptake (**c**). For microscopy images (insets), MOI = 100 and scale bars (bottom right) are 50 µm. Quantification of cell association was calculated as the SYTO signal bound to washed RAW cells divided by the total input SYTO signal (of unwashed *Ft* and cells) at each MOI. * t test *p* < 0.05. Results are combined from 4 independent experiments; means shown with standard deviation (SD). Un-stained *Ft* (- and YFC) were incubated with RAW cells at MOI = 0 (“no *Ft*”) or 500 for 2 h at 4 °C (**b**) or at 37 °C (**d**). Following washes to remove un-bound bacteria, cell-associated material was probed by western blot with Ab specific for the *Ft* protein FopA and the cellular protein β-actin.

Having determined biochemically that YFC was surface-exposed, we next sought evidence that the murine C3d moiety was functional in promoting binding to CR+ cells. *Ft* is an intracellular bacterium that can enter many cells (including MΦs) through multiple receptors (including CR3), depending on which opsonins are present [41,42]. We reasoned that *Ft* genetically decorated with functional C3d should more avidly associate with murine MΦs than *Ft* lacking C3d. To this end, we incubated *Ft*:YFC or *Ft*:- with CR3+ RAW 264.7 MΦs in the absence of serum for 2 h at 4 °C or 37 °C. The experiments were conducted with fluorescently-stained *Ft* (followed by detection of MΦ fluorescence―Figure 2a,c and Figure S3) or with unstained *Ft* (followed by the detection of the *Ft* protein FopA in MΦs―Figure 2b,d). By fluorescence microscopy, MΦs incubated with *Ft*:YFC at 4 °C had a faint, yet elevated, fluorescence above that of MΦs incubated with *Ft*:- (Figure 2a insets and Figure S3). For 37 °C incubations, MΦ fluorescence from *Ft*:YFC was markedly increased above the *Ft*:- controls and it was characterized by many discrete intracellular puncta (Figure 2c insets and Figure S3), consistent with the role of CR3 as the major phagocytic receptor for C3 opsonized *Ft* [43]. These microscopy impressions were confirmed by quantifying MΦ-bound and total (bound plus unbound) fluorescence over a range of multiplicity of infection (MOI) with a plate-reading fluorometer (Figure 2a,c). In these assays, we observed that significantly higher percentages of *Ft*:YFC bacteria remain cell-associated when compared to the empty vector controls. Additional MΦ-binding experiments with un-stained *Ft* were analyzed by western blot for the bacterial and cellular proteins FopA and β-actin (Figure 2b,d). These results further confirmed the notion that the C3d moiety in YFC functions to enhance binding and apparent phagocytosis.

### 2.3. YFC-Bearing Bacteria Induce Elevated p38 and p65 Phosphorylation in Murine Macrophages

**Figure 3:**
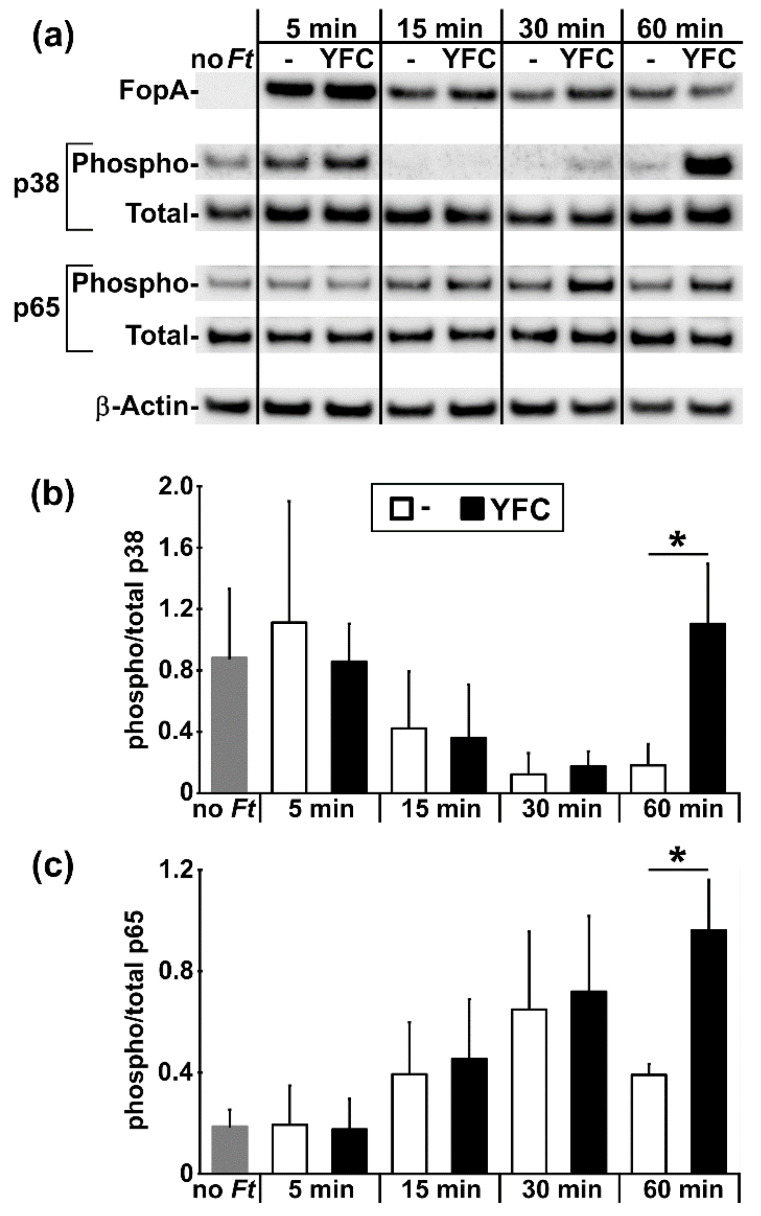
YFC-bearing bacteria induce elevated p38 and p65 phosphorylation in MΦs. (**a**) RAW 264.7 cells were incubated without (“no *Ft*”) or with *Ft*:- or *Ft*:YFC (MOI = 500) at 37 °C for the indicated time. Washed cells were probed by western blot with Abs specific for the indicated proteins. (**b**,**c**) Densitometry of phosphorylated and total forms of p38 and p65 combined from ≥3 independent experiments. * t test *p* < 0.05.

We next sought to determine whether YFC-expressing bacteria elicit distinct cellular responses. Multiple groups have reported that MΦs interacting with wildtype, unopsonized *Ft* produce a transient spike in MAPK phosphorylation, which wanes to baseline as NF-κB activity subsequently peaks and resolves [44,45,46,47]. In recent studies of C3-opsinized *Ft* and CR3 engagement, Dai et al. found that, shortly after phagocytosis, murine MΦs respond to C3-opsonized *Ft* with increased MAPK phosphorylation [43]. Accordingly, we predicted that YFC-expressing bacteria would also induce altered patterns of p38 and NF-κB (p65) phosphorylation. We incubated *Ft*:- and *Ft*:YFC in serum-free media with RAW 264.7 cells, which were harvested at short intervals for western blot analysis (Figure 3a). Shortly following *Ft* infection, the P-p38 levels decreased while the P-p65 levels increased with no consistent differences between control and YFC-expressing *Ft*. However, at the later time points, cells that were infected by *Ft*:YFC contained levels of P-p38 and P-p65 significantly higher than in *Ft*:- infected cells (Figure 3).

### 2.4. Infection of Mice with YFC-Bacteria Transiently Limits Weight Gain and Induces Higher Titers of Ab

**Figure 4:**
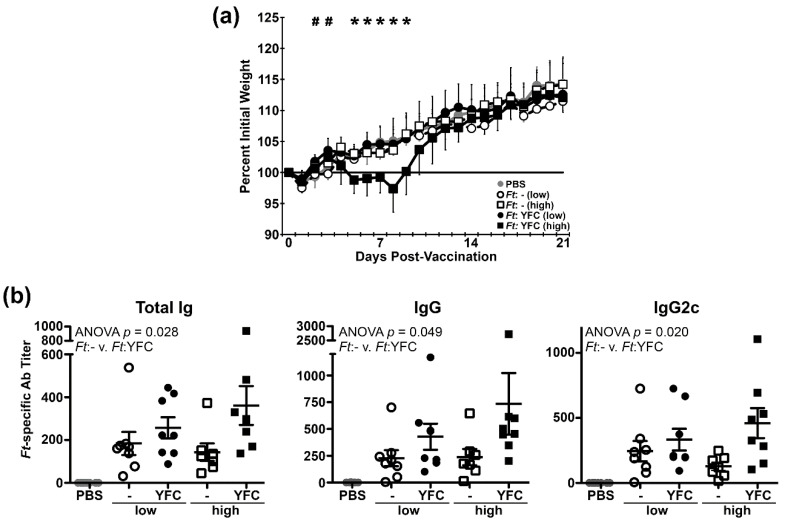
Infection with YFC-bacteria induces higher titers of serum Ab. (**a**) Individual mouse weights recorded daily and expressed as a percentage of their baseline weight (average of d –3–0). 7–8 mice per group. **#** indicates t test *p* < 0.01 “-” vs YFC, low doses. * indicates t test *p* < 0.01 comparing the high doses. (**b**) Sera were collected on d21 post-vaccination and *Ft*-specific titers were determined via ELISA. Two-way ANOVA was used to assess the effect of YFC. 7–8 mice per group.

As the C3d portion of YFC appeared to be functional in cell-based assays, we next sought to determine whether YFC-bearing bacteria would provoke altered responses in animals. In addition to CR3 on MΦs, YFC-expressing bacteria in vivo could potentially engage CR3+ dendritic cells (DCs—potent Ag-presenting cells), CR2+ FDCs, and/or CR2+ B cells. Interaction with the latter cells by C3d-linked moieties has been shown to significantly enhance Ab responses. Accordingly, we first sought to determine whether YFC-expressing bacteria are safe in vivo and if they induced altered Ab responses. To this end, we vaccinated mice intranasally (i.n.) with one of two low doses (~50 and ~ 200 CFU) of live *Ft* LVS bearing either the empty vector (-) or the YFC-expression plasmid. Mice that were vaccinated with the higher dose of *Ft*:YFC exhibited slight weight loss on d ~ 5–9, whereas the remaining groups steadily gained weight following vaccination; all animals appeared to be healthy and survived (Figure 4a). By ELISA, we determined the d 21 serum Ab titers against wildtype *Ft* LVS lysates. We observed significantly increased titers of total Ig against *Ft*, which included total IgG and the IgG2c isotype, among mice that were vaccinated with *Ft*:YFC (Figure 4b). IgM trended towards increased levels in *Ft*:YFC vaccinated mice (*p* = 0.16); IgG1 and IgA were not uniformly detected in either vaccine group (not shown). 

### 2.5. Vaccination with YFC-Bacteria Alters Ag Recognition and Limits Morbidity Following Challenge 

**Figure 5:**
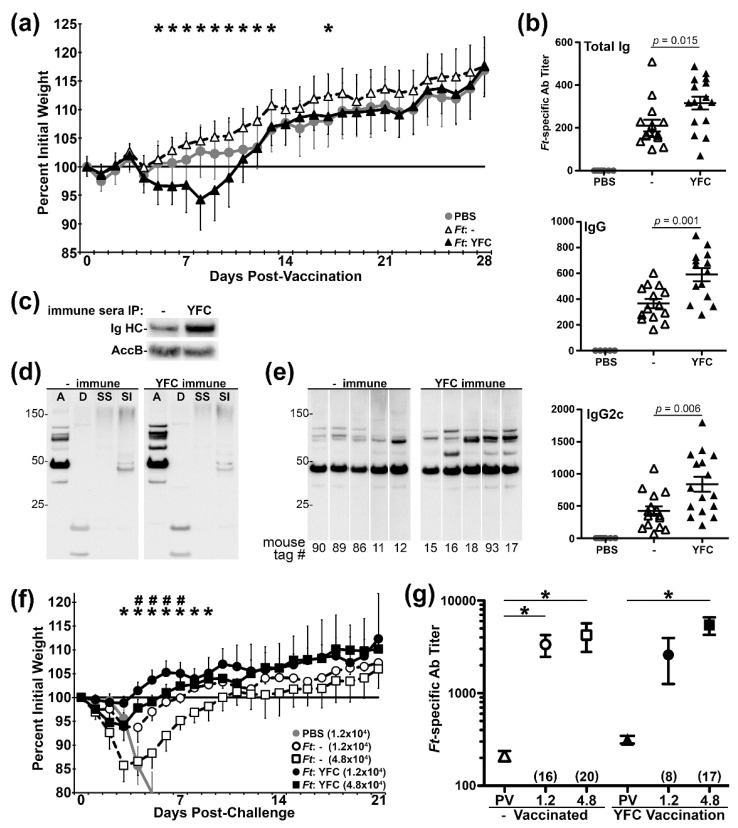
Vaccination with YFC-bacteria alters Ag recognition and limits challenge-induced morbidity. (**a**) Individual mouse weights recorded daily and expressed as a percent of their baseline weight (average of d –3–0); 8 PBS mice and 15–16 mice per vaccine group. * indicates t test *p* < 0.01 comparing *Ft*:- and *Ft*:YFC. (**b**) Sera collected 21 d post-vaccination (PV) were assessed by ELISA for *Ft*-specific Ig, IgG, and IgG2c titers. *P* values derived from t tests of “-” vs YFC. (**c**) PV sera pooled from “-” or YFC vaccinated mice were used at equal dilution to test IP with *Ft* Live Vaccine Strain (LVS). Washed bacteria were probed via western blot for Ig HC. (**d**) PV sera pooled from mice vaccinated with “-” or YFC were used at equal titers to probe by western blot aqueous (A), detergent (D), sarkosyl soluble (SS), and sarkosyl insoluble (SI) phases of WT *Ft* LVS. (**e**) PV sera from individual mice vaccinated with “-” or YFC were used at equal titers to probe by western blot A phases of WT *Ft* LVS. (**f**) The mice were challenged i.n. with 12k or 48k CFU of WT *Ft* LVS on d 28 PV. Individual post-challenge mouse weights recorded daily and expressed as a percent of their baseline weight (average of d 26–28 PV) with 4–6 mice per group. **#** t test *p* < 0.01 between “-” and YFC at the 12k dose. * t test *p* < 0.01 between “-” and YFC at the 48k dose. (**g**) Sera collected on d 21 PC were analyzed by ELISA along with PV sera for total *Ft*-specific Ig titers. * ANOVA with Tukey post-test *p* < 0.01 between indicated groups. Fold-increase (PC/PV) of average titer for each group is indicated in parentheses.

Having established that *Ft*:YFC was well tolerated and induced elevated Ab responses, we next vaccinated mice i.n. with a single dose (~200 CFU) of *Ft*:- and *Ft*:YFC to further characterize vaccine-induced responses. Among *Ft*:YFC immunized mice, we again observed a slight decrease in weight following vaccination (Figure 5a) and significant increases in *Ft*-specific serum Ig, IgG, and IgG2c titers (Figure 5b). We used live wildtype *Ft* to IP serum Ab and detected bound Ig heavy chain by western blot to determine whether the *Ft*:YFC-induced sera also contained more opsonizing Ab. Sera derived from *Ft*:YFC vaccinated mice contains more bacterial surface-binding Ab than does sera from control immunized mice. We also used these sera pools to probe fractionated *Ft* (aqueous [A], detergent [D], sarkosyl soluble [SS], sarkosyl insoluble [SI] phases) via western blot to gauge the repertoire of bacterial Ags recognized, as shown in Figure 5c. For these assays, the blots were probed with equal titers of the two sera and, as expected, the majority of bacterial Ags were equivalently recognized (Figure 5d). However, a small sub-set (~3–4) of A-phase proteins between ~50–100 kDa were more robustly recognized by the *Ft*:YFC immune sera. When sera from individual animals were similarly used at equal titer, we again observed elevated reactivity among *Ft*:YFC-immunized mice for these 3–4 A-phase Ags (Figure 5e). Collectively, *Ft*:YFC immunized mice produce higher titers of serum Ab that contains more opsonizing Ig and recognizes a broader repertoire of bacterial Ags. Next, these mice were challenged i.n. with one of two doses (12k or 48k CFU) of wildtype *Ft* LVS. These doses were known to be lethal for naïve controls (LD_50_ = 1250 CFU), but expected to be sublethal for LVS immunized mice. Indeed, the PBS controls succumb to challenge by d 9 and all immunized mice ultimately survived. However, differences were apparent in the magnitude and duration of challenge-induced morbidity—as indicated by weight loss. At both challenge doses, *Ft*:YFC immunized mice lost less weight than their *Ft*:- counterparts and the differences remained significant for 4–7 d (Figure 5f). When the days below baseline weight were calculated, these were also significantly lower for *Ft*:YFC immunized mice. Among the 12k CFU challenge animals, the *Ft*:YFC immunized mice were below baseline weight for 2.3 +/- 1.5 d following challenge as compared to 6.2 +/- 0.8 d for the *Ft*:- vaccinated group (*p* = 0.001). For the 48k challenge recipients, the corresponding numbers were 5.0 +/- 0.8 d for the *Ft*:YFC vaccinated mice and 13.8 +/- 5.1 d for the control *Ft*:- mice (*p* = 0.01). Finally, we compared post-vaccination (PV) and post-challenge (PC) serum Ab titers among these groups and made an unexpected observation. *Ft*:YFC vaccinated mice, which had higher PV titers and less challenge-induced morbidity, appeared to have a muted Ab response to challenge. For *Ft*:- immunized mice, we observed 16- and 20-fold increases (PC/PV) in mean serum Ig titers for the 12k and 48k doses, respectively (Figure 5g). For *Ft*:YFC immunized mice, the fold changes were 8 and 17. 

### 2.6. Vaccination with YFC-Bacteria Accelerates Recovery Following Challenge with a Heterologous Isolate

**Figure 6:**
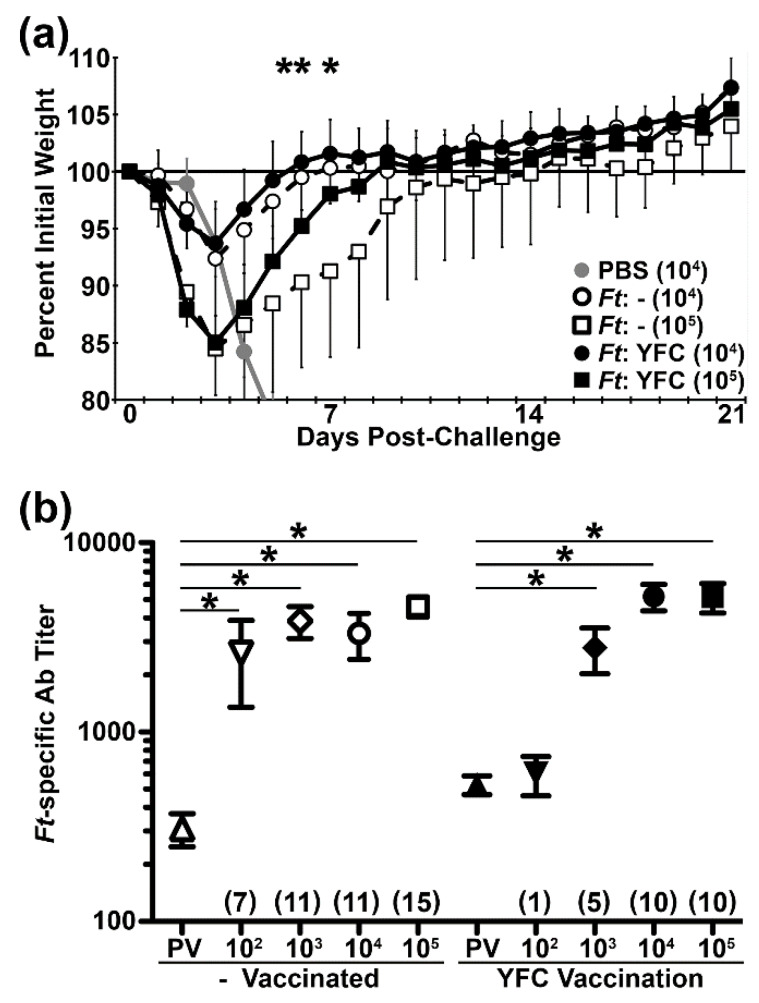
Mice vaccinated with YFC-bacteria display improved weight-gain following heterologous challenge. (**a**) *Ft*:- and *Ft*:YFC vaccinated mice were challenged i.n. with *Ft* LVS RML. Individual post-challenge mouse weights are expressed as a percent of their baseline weight (average of d 26–28 PV) with 7–8 mice per group. * t test *p* < 0.05 between “-” and YFC for 10^5^ challenge dose. (**b**) Sera collected on d 21 PC were analyzed by ELISA along with PV sera for total *Ft*-specific Ig titers. * ANOVA with Tukey post-test *p* < 0.05 between indicated groups. Fold-increase (PC/PV) of average titer is indicated in parentheses.

In the preceding work, one isolate of *Ft* LVS was the basis of the vaccine strains and the challenge agent. As virulence can vary among isolates, we sought to determine whether our *Ft*:YFC strain could similarly protect against challenge with Rocky Mountain Laboratory (RML) *Ft* LVS, which is ~ 50x more virulent than ATCC LVS [48]. We immunized four groups of mice with our *Ft*:- or *Ft*:YFC vaccine strains. The *Ft*:YFC immunized mice again displayed transient weight loss following vaccination (not shown) and significantly increased the serum Ab titers PV (not shown). The vaccinated mice were then challenged i.n. with 10^2^, 10^3^, 10^4^, or 10^5^ CFU of RML *Ft* LVS. PBS control mice were challenged i.n. with 10^2^, 10^3^, or 10^4^ CFU of RML *Ft* LVS. Survival among PBS controls was 100%, 37.5%, and 0%, respectively; vaccinated mice ultimately survived all challenge doses (Figure S4). We again noted significant morbidity differences between *Ft*:- and *Ft*:YFC immunized mice (Figure 6a), albeit with some distinctions. In contrast to the homologous challenge results, here the magnitude of peak weight loss did not differ between *Ft*:- and *Ft*:YFC immunized mice. However, the latter did recover weight more quickly (Figure 6a) in ordeer to yield a trend towards fewer days underweight. Among the 10^5^ CFU challenge animals *Ft*:YFC immunized mice were below baseline weight for 8.8 +/- 2.9 d after challenge when compared to 12.6 +/- 6.0 d for the *Ft*:- vaccinated group (*p* = 0.13). Last, we compared PV and PC serum Ab titers and again observed that mice immunized with *Ft*:YFC required much higher challenge doses to provoke a significant Ab-recall response (Figure 6b).

## 3. Discussion

We sought to simplify vaccine development through generation of a plasmid-encoded AT-CRL fusion. The transformation of the platform into gram-negative bacteria results in the expression of a CRL (C3d or p28) on the bacterial surface; consequently, targeting them to CRs on immune cells. Engagement of CR3 on MΦs induces phagocytosis, signaling, and entrance into peripheral lymph nodes [49]. CR2 engagement on B cells and FDCs is also an important aspect of forming an adaptive immune response—inducing prolonged Ag display in follicles, uptake by and activation of B cells, and increased Ab responses [50].

In this work, we have modified trimeric AT YadA of *Ye* to include a CRL, yielding constructs YFP and YFC (Figure 1). The expression and trimerization of these constructs in *Ec*, *Kp*, and *Ft* varied. Reduced trimeric-sized bands that were observed in *Ft*:YFC compared to *Ec*:YFC and *Kp*:YFC are likely not indicative of a lack of trimer formation in vivo since detectable surface exposure is observed, but rather might be due to the reduced stability of the protein when boiled in reducing sample buffer and resolved via SDS-PAGE. However, reduced surface accessibility of the constructs in some strains might be due to lack of proper chaperone complexes and/or interference by host proteases preventing proper localization, trimerization, and/or transport of passenger domains through the β-barrel. In future work, we will aim to improve the broad utility of our constructs by co-expressing chaperones, such as *Ye* Skp, which aids in the heterologous expression of YadA [51].

Utilizing *Ft* as a model, we have shown that YFC expression in bacteria increases association with CR3-expressing macrophages (Figure 2). Previous studies support that this increased interaction is likely due to CRL engagement of CR3 on these cells [41,43], although we have not formerly defined the receptor specificity here. The engagement of TLRs on the surface of these cells is likely also an important aspect of CR3 binding and induction of phagocytosis. TLRs, specifically TLR2 in the case of *Ft*, initiate inflammatory signaling and inside-out signaling through phosphatidylinositol-3-kinase [43,52,53,54]. This inside-out signaling results in a conformational change of CR3 from closed to open/active, allowing for CRL engagement [55,56]. In addition to increased binding, we have also observed an increased phosphorylation of p38 and p65 in MΦs at 60 min. post-incubation with *Ft*:YFC (Figure 3). This largely supports work by Dai et al., who showed similar increases in inflammatory signaling when C3-opsonized *Ft* SchuS4 were incubated with murine MΦs; contrasted by their similar experiments in human macrophages, which resulted in the inhibition of this inflammatory signaling [43]. 

The increased signaling we observe could also be due to intracellular detection of C3d [57]. The pathway by which this detection occurs is not fully understood. Some studies have shown that the detection of cytoplasmic C3b by mitochondrial antiviral-signaling protein (MAVS) induces NF-κB pathways, which is consistent with the increased p65 phosphorylation that we observed [58,59,60,61]. Additionally, other groups have found that the intracellular C3b protein induces the formation of autophagosomes and increases the degradation of the accompanying pathogen [58,62], a phenomenon that might also contribute to intracellular pathogen spread via trogocytosis [63] and has been suggested as a virulence mechanism for *Ft* [64,65,66]. In contrast, *Ft* has also been shown to evade autophagy host defenses [67]. Further experiments are required in order to elucidate the exact mechanism at play within our system; we propose that assessing MΦ health as well as *Ft*:YFC viability and/or replication in MΦs over time will contribute to our understanding. 

We administered *Ft*:YFC and *Ft*:- strains to C57BL/6 mice i.n. in order to assess the effect of YFC on the immune response in vivo. On d 21 PV, we observed increased pathogen-specific Ab titers as well as increased diversity in proteins recognized compared to the *Ft*:- control (Figure 4 and Figure 5). This promising result agrees with previous works that have shown similar titer increases when fusing C3d to a subunit vaccine; however, our approach involves Ab development against a wide array of bacterial Ags rather than a solitary peptide. *Ft*:YFC vaccinated mice also exhibited protection against *Ft* LVS challenge-induced weight loss (Figure 5f), which suggested that the increased immune response generated translates to an effective response against subsequent pathogen exposure. We assessed whether this protection would translate to protection against challenge with a heterologous, more virulent isolate of *Ft,* the RML strain [48]. Again, we observed that *Ft*:YFC vaccinated mice exhibited a faster recovery from challenge-induced weight loss (Figure 6a). In order to assess the effectiveness of our CR-targeted vaccine strain at preventing lethality, we would likely need to use a type A strain of *Ft*, such as SchuS4, for a highly stringent challenge. Moreover, additional assays would be required to reveal the relative contributions of the humoral and cellular responses to *Ft*:YFC.

An unexpected observation came from the comparisons among the PV and PC Ab titer determinations. Specifically, the finding that *Ft*:YFC immunized mice, which had higher PV titers, appeared to have a muted Ab response to challenge (Figure 5g and Figure 6b). This, along with protection from challenge-induced weight loss, supports the notion that higher Ab titers prior to challenge is associated with decreased disease severity and a diminished need for a prominent recall response at low challenge doses; a phenomenon that has been observed in humans [68,69,70,71,72]. Subjects with higher pre-existing Ab titers against flaviviruses [68], *Shigella* [70,72], and influenza [69,71] experience reduced disease upon respective infection and reduced Ab recall response. Therefore, we believe that our results are likely translatable to other diseases as well as human models of infection. 

Our advancement in *Ft* vaccine development is important, as there is currently no FDA-approved vaccine and human-virulent strains of *Ft* that have been developed as biological weapons [73]. Furthermore, we have also successfully expressed AT-CRLs in *Ec* and *Kp (*Figure 1e–g). Preliminary results support surface exposure of our platform in these other gram-negative bacteria and offer exciting applicability to pathogens that are increasingly becoming antibiotic resistant [74]. It would be interesting to test the efficacy of targeted *Ec*:YFP, *Ec*:YFC, and *Kp*:YFP strains in cell-based and animal studies in *Ec* and *Kp* models of infection. Based on differences between gram-negative and gram-positive cell walls, AT-CRL would not be applicable to gram-positive bacteria. However, we could envision expanding this targeting approach to vaccine development against additional gram-negative pathogens, as the AT-CRL approach does not require pre-existing pathogen-specific Abs, identification of protective antigens, or addition of adjuvants in order to enhance immune responses. Finally, the potential broad-applicability of our platform also offers an interesting opportunity for combination with pre-existing targeting strategies, such as FcR targeting, to further advance vaccine development. 

## 4. Materials and Methods 

### 4.1. Bacteria

*Escherichia coli* (*Ec*) One Shot^TM^ TOP10 Chemically Competent (Invitrogen, Carlsbad, CA, USA) cells were used as the host strain for plasmid construction. *Ec* were cultured in Luria-Bertani (LB) broth or on LB agar plates with appropriate antibiotics at either 26 °C or 37 °C where indicated. Electro-competent *Francisella tularensis* (*Ft*) LVS NR646 (BEI Resources, Manassas, VA, USA) and *Klebsiella pneumoniae* (*Kp*) ATCC 13883 (provided by Robert K. Ernst, Ph.D.) were produced as follows: overnight cultures were harvested (prior to reaching an OD at 600 nm of 0.6) via centrifugation at 4000 g for 15 min. at 4 °C. Cells were placed on ice and all solutions were chilled. Cells were re-suspended in 0.5 M sucrose, spun, and again re-suspended in a smaller volume of 0.5 M sucrose until a final volume of 500 µL was reached. The cells were then aliquoted and utilized for electroporation or frozen for later use. *Ft* strains were cultured at 37 °C in Brain Heart Infusion (BHI) media (pH 6.8) with appropriate antibiotics or on BHI agar plates that were supplemented with 2.5% L-cysteine hydrochloride, 5% hemoglobin, and appropriate antibiotics. *Kp* were cultured in LB broth or on LB agar plates with appropriate antibiotics at 26 °C.

### 4.2. Plasmids

The pCR 2.1-Topo vector and TOPO TA Cloning Kit (Invitrogen) were used for direct cloning of PCR products. The broader-host-range shuttle vectors pF and pF2, which encode kanamycin resistance, were used to express YadA, YFC, and YFP in *Ec*, *Kp*, and *Ft.* The pF/F2 plasmids include a M13R priming site, a *Ft groEL* promoter (truncated in pF2) driving expression of inserts (see below), and a reverse priming site for pF-Reverse. Bacteria that were transformed with empty pF/pF2 vectors served as controls and they are indicated as “**-**” in figures.

YadA. Full length *yadA* was PCR amplified from *Y. enterolitica* DNA with primers Ye YadA 5EcoRI and Ye YadA 3Stop_PmeI and then cloned into pCR 2.1-TOPO to generate pCR 2.1-TOPO:YadA. Table S1 provides primer sequences. The YadA EcoR1/PmeI fragment was digested from pCR 2.1-TOPO:YadA, gel-purified, and then ligated into EcoRI/Sma digested pF or pF2 yielding pF:YadA and pF2:YadA (designated as “YadA” in figures).

pF:YadA SS-SβB. Two fragments of *yadA* were PCR amplified from *Y. enterolitica* DNA and separately cloned separately into pCR 2.1-TOPO prior to DNA sequence analysis. The first fragment, encoding the YadA signal sequence (YadA SS), was amplified while using primers Ye YadA 5EcoRI and 3’ YadA SS_SacI. The second fragment, encoding the YadA stalk and β-barrel (YadA SβB), was amplified using primers 5’ SacI_FLAG_YadA C term and Ye YadA 3Stop_PmeI. 5’ SacI_FLAG_YadA C term encodes an engineered, in-frame FLAG tag and Ye YadA 3Stop_PmeI contains an additional in-frame stop codon. Following digestion of pCR 2.1-TOPO:YadA SβB with EcoRI and PmeI, YadA SβB was gel purified and then ligated to EcoRI/EcoRV digested pF, resulting in plasmid pF:YadA SβB. YadA SS was liberated from pCR 2.1-TOPO:YadA SS with EcoRI and SacI, gel purified, and then ligated to similarly digested pF:YadA SβB. Salient features of the resulting plasmid (pF:YadA SS-SβB) include an open-reading frame containing the YadA SS, a SacI site, a FLAG-tag and YadA SβB followed by 2 stop codons. The SacI site in pF:YadA SS-SβB subsequently served as the recipient site for SacI-flanked inserts encoding complement receptor ligands (below).

YFC. DNA encoding murine C3d was PCR amplified from TOPO: C3d rvs BglII BSSB while using 5’ C3d w/linker AgeI and 3’ C3d w/linker XmaI_SalI and cloned into pCR 2.1-TOPO prior to DNA sequence analysis. The resulting plasmid was subject to site directed mutagenesis (QuikChange Site-Directed Mutagenesis Kit, Agilent Technologies, Santa Clara, CA, USA) with 5’ Mut. Destroy SacI in C3d and 3’ Mut. Destroy SacI in C3d in order to eliminate the C3d-internal SacI site. The resulting mutated C3d DNA was PCR amplified with 5’ SacI_C3d and 3’ C3d_SacI and cloned into pCR 2.1-TOPO yielding pCR 2.1-TOPO:C3d-NoIntSacI. Following the digestion of pCR 2.1-TOPO:C3d-NoIntSacI with SacI, the C3d-NoIntSac fragment was gel purified and then ligated to SacI digested pF:YadA SS-SβB (above). A clone with the C3d fragment in the same orientation as YadA was identified and termed YFC.

YFP. DNA encoding murine p28, flanked by SacI sites, was generated via synthetic overlap PCR using primers P28 FWD SacI (for YadA) and P28 RVS SacI (for YadA) and it was cloned into pCR 2.1-TOPO yielding pCR 2.1-TOPO:SacI-p28-SacI. pCR 2.1-TOPO:SacI-p28-SacI was digested with SacI and the p28 fragment was then gel purified and ligated to SacI-digested pF:YadA SS-SβB (above). A clone with the p28 fragment in the same orientation as YadA was identified via DNA sequencing and it was termed YFP. 

### 4.3. SDS-PAGE and Western Blots

Bacterial whole cell preps (in TPIG = 50 mM Tris 8.0, protease inhibitor cocktail [Sigma-Aldrich, St. Louis, MO, USA. P2714], 10% glycerol) were boiled in Laemmli sample buffer for 10 min. and were loaded at 10^8^ cells per lane onto NuPAGE 4–12% Bis-Tris Protein Gels (Invitrogen). The NuPAGE MES SDS running buffer was used and gels were run variously at 50–120 V. Resolved gels were transferred to 0.22 µm nitrocellulose membranes (BioRad, Hercules, CA, USA). Membranes were blocked for 30 min. in 5% non-fat dry milk in PBS 0.05% Tween 20 (PBST); except when anti-goat antibodies were used, in which case membranes were blocked for 30 min. with 5% heat-inactivated FBS in PBST. Primary antibodies were used at the indicated concentrations for an overnight incubation at 4 °C in PBST. Secondary horseradish peroxidase (HRP)-conjugated antibodies were diluted to the indicated concentrations in PBST and incubated at 4 °C for 1 h. Where indicated, a biotinylated secondary antibody was used, followed by streptavidin-linked HRP, each for 1 h incubations at 4 °C. The development of chemiluminescent substrate (SuperSignal West Pico or SuperSignal West Femto Maximum Sensitivity, Pierce) was visualized using the BioRad ChemiDoc Touch Imaging System. Densitometry was analyzed using the BioRad Image Lab 6.0 software. 

### 4.4. Bacterial Fractionation

Whole cell preps of bacteria were incubated at 2.5 × 10^7^ cells/µL in 1% Tx114 (containing protease inhibitor (Sigma-Aldrich, P2714), lysozyme (Fisher scientific, Waltham, MA, USA, BP535), and benzonase nuclease (Sigma-Aldrich, E8263) in PBS) for 30 min. at room temperature. The samples were then placed on ice for 30 min. and subsequently centrifuged at 13,000 g for 30 min. at 4 °C and the Tx114 soluble (TxS) phase was separated from the Tx114 insoluble (TxI) phase. TxI was resuspended in PBS and both TxI and TxS were again centrifuged; residual TxS was removed from TxI and TxS was moved to a fresh tube to remove any residual TxI. TxS was then incubated at 31 °C for ~5 min. to precipitate Tx114, and the samples were centrifuged at room temperature for 10 min. at 10,000 g in order to separate aqueous (A) and detergent (D) soluble fractions. A and D were both washed three times. D was re-suspended in 1% SDS to a final concentration of 10^8^/µL. TxI was also further processed and it was re-suspended in 0.2% sarkosyl and then incubated at room temperature for 30 min. The TxI samples were then centrifuged at 13,000 g for 30 min. at 4 °C and the sarkosyl soluble (SS) phase was removed from the sarkosyl insoluble (SI) phase. Extraction from SI was repeated via 0.2% sarkosyl incubation and centrifugation. SI was then re-suspended in PBS and both SS and SI were again centrifuged to remove residual SS from SI and residual SI from SS. SS was transferred to a fresh tube. SI was re-suspended in 1% SDS to a concentration of 10^8^/µL. All of the samples were loaded at equivalents to 10^8^ bacteria per lane for SDS-PAGE. 

### 4.5. Collagen Binding ELISA

Flat bottom Costar 96-well assay plates (Corning #9018) were coated with 1µg of type I collagen (rat tail, Sigma-Aldrich, C3867) in 100 µL of 100 mM bicarbonate/carbonate coating buffer (pH 9.6) per well, incubating overnight at 4 °C. The wells were washed three times with PBS 0.05% Tween 20 while using the BioTek ELx50 plate washer and then blocked for 2 h at room temperature with 2% heat inactivated (HI) FBS in DPBS. *Ft* strains were serially diluted in blocking solution (2% HI FBS in DPBS) and then incubated on the collagen-coated plate for 1 h at room temperature. The plates were then washed and *Ft* was detected using 1:2500 Mouse anti-*Ft* LPS (clone FB11, Abcam, Cambridge, UK) followed by 1:5000 Goat anti-Mouse IgG(H+L) HRP (Southern Biotech, Birmingham, AL, USA); each diluted in blocking buffer and then incubated for 1 h at room temperature. Tetramethylbenzidine (TMB, Sigma-Aldrich) substrate was added, development was stopped using 2 M H_2_SO_4_, and absorbance was measured at 450 nm while using the BioRad iMark microplate reader.

### 4.6. Antibody Immunoprecipitation

25 µL of bacteria in culture media (at 2.5 × 10^7^ CFU/µL) were mixed with 25 µL of the appropriate antibody in culture media and then incubated for 1 h at room temperature. Mouse α-FLAG, biotinylated (Sigma-Aldrich, F9291) was used at a final concentration of 6 µg/µL. Rat α-complement C3 (Novus NB200-540) was used at a final concentration of 12 µg/µL. Mouse α-IglC (clone 1G7 hybridoma supernatant) [75] was used at a 1:45 dilution. The samples were centrifuged at 5000 g for 10 min., washed with fresh culture media, and re-suspended to 10^7^/µL in TPIG before prep for western blotting. 

### 4.7. Cell Culture 

RAW 264.7 cells were maintained at 37 °C in 5% CO_2_ in DMEM (high glucose, Gibco, Gaithersburg, MD, USA) that was supplemented with 10% heat inactivated fetal bovine serum, 100 units/ml penicillin-streptomycin (Gibco), 2 mM GlutaMAX (Gibco), 10 mM HEPES (Gibco), 0.075% sodium bicarbonate (Gibco), and 50 µM 2-mercaptoethanol (Sigma-Aldrich). For co-incubations of RAW cells with bacteria: serum-, antibiotic-, and mercaptoethanol-free (SAM-free) formulations were used. 

### 4.8. Bacteria Staining and Quantification of RAW 264.7 Cell Binding 

For microplate-based quantification: RAW 264.7 cells were seeded on Greiner CELLSTAR black-wall 96-well plates, aiming for confluency following overnight incubation. BHI-grown *Ft* were stained by incubating the bacteria with 5 µM of SYTO BC Green Fluorescent Nucleic Acid Stain (Invitrogen, S34855) in DPBS at 10^7^*Ft*/µL, *Ft* were then washed and re-suspended in SAM-free DMEM. *Ft* strains were serially diluted in SAM-free DMEM, starting at 10^8^/well (~MOI 1000) in 100 µL, and they were incubated with RAW cells for 2 h at either 4 °C or 37 °C. The wells were washed with SAM-free DMEM before adding DPBS and measuring fluorescence with the PerkinElmer VICTOR Nivo Microplate Reader. Output was plotted as a percentage of the input *Ft* signal. For Microscopy: RAW cells were seeded on MatTek 24-well No. 1.5 Coverslip plates and they were incubated with stained (as above) *Ft* strains at MOI 100 in 1 mL SAM-free DMEM for 2 h at either 4 °C or 37 °C. Wells were washed and imaging was performed at 40x with the Zeiss Axio Observer. For RAW cell incubation with unstained *Ft* strains: RAW cells were seeded on Corning Costar 24-well plates, aiming for confluency overnight, and they were incubated with 5 × 10^7^ *Ft*/well (~MOI 500) in 500 µL SAM-free DMEM for 2 h at either 4 °C or 37 °C. The wells were washed with SAM-free DMEM and adherent cells were harvested into 150 µL of 0.5% Tx100 and combined with 150 µL Laemmli sample buffer in preparation for western blotting. 

### 4.9. RAW 264.7 Cell Signaling Experiments

RAW 264.7 cells were seeded on Corning Costar 24-well plates, aiming for confluency overnight and 5 × 10^7^ *Ft*/well (~MOI 500) in SAM-free DMEM were added. The plates were centrifuged at 500 g for 1 min. to synchronize bacterial association with RAW cells and they were incubated at 37 °C for 5, 15, 30, or 60 min. The wells were washed and all cells were collected with 150 µL of 0.5% Tx100 and were added to 150 µL of Laemmli sample buffer in preparation for western blotting for various cell signaling proteins. 

### 4.10. Mice and Immunizations

The Institutional Animal Care and Use Committees of Albany Medical Collage approved (ACUP # 17-09003) the work described in this manuscript. Six-week old male and female C57BL/6 mice (Taconic) were anesthetized via intraperitoneal injection of a cocktail of ketamine (5 mg/kg) (Vedco Inc, St. Joseph, MO, USA) and xylazine (4 mg/kg) (Akorn Inc, Lake Forest, IL, USA) and checked for loss of toe pinch reflex. The anesthetized mice were vaccinated intranasally (i.n.) with a P20 micropipette on d 0 with 20 μL of PBS, *Ft*:-, or *Ft*:YFC drop-wise into a single nare using PBS as a vehicle for *Ft* strains [17,18]. Prior to administration, the *Ft* doses were spotted on agar plates, which were incubated at 37 °C for enumeration of actual CFU counts. Figure 4: intended doses: low = 50 CFU, high = 200 CFU. Actual doses: *Ft*:- (low) = 37 CFU, *Ft*:YFC (low) = 18 CFU, *Ft*:- (high) = 144 CFU, *Ft*:YFC (high) = 36 CFU. Figure 5: intended dose = 200 CFU. Actual doses: *Ft*:- = 159 CFU, *Ft*:YFC = 117 CFU. Figure 6: intended dose: 200 CFU. Actual doses: *Ft*:- = 187 CFU, *Ft*:YFC = 197 CFU. Mice were individually weighed daily starting d –3. Weights are plotted as a percentage of an individual three-day-average baseline. On d 21, blood was collected from the sub-mandibular vein of all mice for post-vaccination (PV) antibody titer analysis. On d 28, mice were anesthetized and challenged i.n. with 20 μL of *Ft* LVS into a single nare. Figure 5: intended low challenge dose = 12,000 CFU, actual = 12,500 CFU. Intended high challenge dose = 48,000 CFU, actual = 55,500 CFU. Figure 6: intended *Ft* LVS RML doses: 10^2^, 10^3^, 10^4^, 10^5^ CFU. Actual doses: 133, 1194, 9844, 107,813 CFU. Mice were followed for 21 d post-challenge (PC) and individual weights were recorded. Figure 5 and Figure 6: blood was collected from the sub-mandibular vein of all mice for post-challenge antibody titer analysis on d 21 PC. 

### 4.11. Antibody Titer Determination

Sera were prepared from PV and PC blood collections by centrifuging clotted blood at 2000 g for 8 min. and was then analyzed for *Ft*-specific titers via ELISA. Flat bottom Costar 96-well assay plates (Corning #9018) were coated with 100 µL of *Ft* LVS whole cell lysate derived from 5 × 10^5^ *Ft*/well in coating buffer (100 mM bicarbonate/carbonate coating buffer pH 9.6) overnight at 4 °C. Wells were washed three times with PBS 0.05% Tween 20 using the BioTek ELx50 plate washer and were then blocked with 2.5% heat inactivated (HI) FBS in DPBS. The plates were again washed and incubated with serial dilutions of immune sera in blocking solution (2% HI FBS in DPBS) for 1 h at room temperature. *Ft*-specific Ab were detected with 1:5000 of the respective goat anti-mouse HRP-conjugated secondary, incubated in blocking buffer for 1 h at room temperature. The plates were again washed, tetramethylbenzidine (TMB, Sigma-Aldrich) substrate was added, development was stopped using 2 M H_2_SO_4_, and absorbance was measured at 450 nm using BioRad iMark microplate reader. Titers were determined as the Ec50s of the nonlinear regression analysis of log transformed values. 

### 4.12. Statistics

GraphPad Prism 5 or Microsoft Excel calculated the statistically significant differences. Differences in fluorescent signal measured by the PerkinElmer VICTOR Nivo Microplate Reader, differences in quantitated densitometry for signaling experiments, differences in mouse weights, and differences in days below baseline weight were all calculated via two-tailed unpaired t-test when comparing the control (-) to YFC. Differences in *Ft*-specific antibody titers post-vaccination were determined by two-way ANOVA (Figure 5) or t-test (Figure 6), when comparing the control (-) to YFC. A comparison of *Ft*-specific antibody titers between post-vaccination and post-challenge sera was determined via one-way ANOVA with a Tukey multiple comparisons test. *p*-value < 0.05 or <0.01 were considered significant where indicated. Ab titer data are presented as the means and standard error of the mean; all other graphical data are means and standard deviations.

## Supplementary Materials

The following will be made available online at https://www.mdpi.com/2076-0817/9/5/375/s1. Table S1: DNAs, bacteria, and primers used in this study. Figure S1: YadA and YFC are outer membrane proteins in *Ft*. Figure S2: Detection of YFC trimers in *Ec* and *Ft*. Figure S3: Expanded Figure 2a,b inset images: YFC enhances association of *Ft* to RAW 264.7 cells. Figure S4: Weight loss following RML challenge.
