# Supplementary material for: Autotransporter-Mediated Display of Complement Receptor Ligands by Gram-Negative Bacteria Increases Antibody Responses and Limits Disease Severity
Source: Pathogens. 2020 May 14;9(5):375. doi: 10.3390/pathogens9050375 (PMC7281241; doi:10.3390/pathogens9050375)
Supplement: Supplementary file 1 [file pathogens-09-00375-s001.pdf]

Supplementary Materials:

Table S1. DNAs, bacteria, and primers used in this study.

**DNAs**	**Source**
pCR 2.1-TOPO	Invitrogen
pF / pF2	Reference [1]
Yersinia enterocolitica ATCC 27729 DNA	Jr-Shiuan Lin, PhD. Trudeau Institute
TOPO: C3d rvs BglII BSSB	USPTO # 9475853
pF/F2 expressing YadA, YFC, or YFP	This study

**Bacteria**	**Source**
Escherichia coli TOP10	Invitrogen
Francisella tularensis LVS (NR646)	BEI Resources
Francisella tularensis LVS (RML)	Douglas Reed, PhD
Klebsiella pneumoniae ATCC 13883	Robert K. Ernst, PhD.

**Primers**	**Sequence**
Ye YadA 5EcoRI	5′ - GAA TTC ACT AAA GAA ATA TAA AAG GTG CTT ACA
Ye YadA 3Stop_PmeI	5′ - GT TTA AAC **CTA** TTA CCA CTC GAT ATT AAA TGA TGC ATT
3′ YadA SS_SacI	5′ - GAG CTC GTC ATT ATT GGC AAA TGC
5′ SacI_FLAG_YadA C term	5′ - GAG CTC GAC TAT AAG GAC GAT GAT GAC AAA TTG GAT ATG GCA AAA AAA CAC TCA AAT AG
5′ C3d w/linker AgeI	5′ - ACC GGT GGG GGG GAA CAG AAC ATG ATT GGC ATG
3′ C3d w/linker XmaI_SalI	5′ – GTC GAC GGA CCC GGG ACC TCC GTT CAA GTC CTT ATG GTC
5′ Mut. Destroy SacI in C3d	5′ - G CAA GAG GCC CTG GAG CTG ATC AAG AAA GGG TAC
3′ Mut. Destroy SacI in C3d	5′ - GTA CCC TTT CTT GAT CAG CTC CAG GGC CTC TTG C
5′ SacI_C3d	5′ - GAG CTC GGG GGG GAA CAG AAC ATG ATT GG
3′ C3d_SacI	5′ - GAG CTC ACC TCC GTT CAA GTC CTT ATG GTC
P28 FWD SacI (for YadA)	5′ - TTG GAG CTC AAG TTT CTG AAC ACA GCC AAA GAT CGG AAC CGC TGG GAG GAG CCT GAC CAG CAG CTC TAC AAC GTA GAG GC
P28 RVS SacI (for YadA)	5′ - TCC GAG CTC GCT GCT GCC ACC TCC TCC GCT GCT CCC ACC TCC CCC GGC GTA GGA TGT GGC CTC TAC GTT GTA GAG CTG CTG

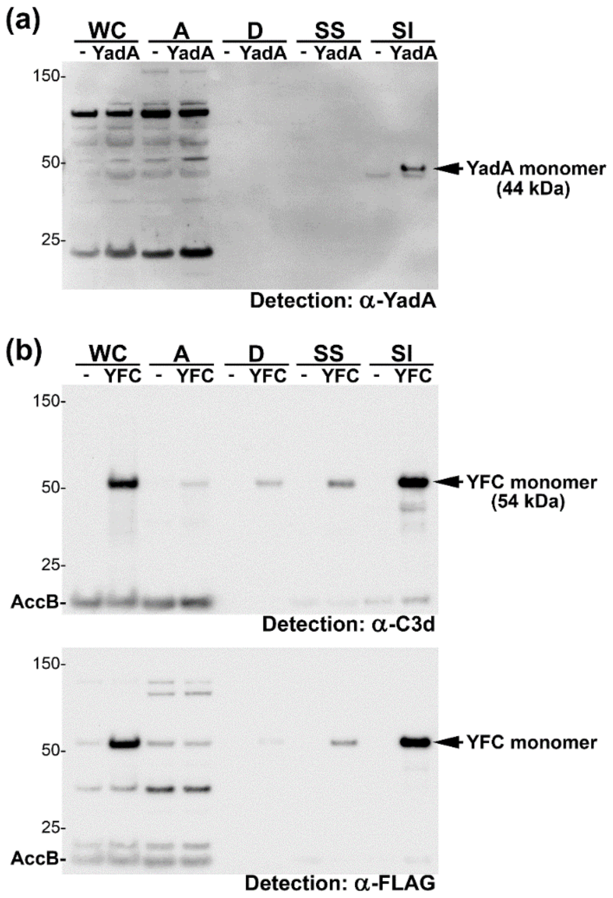

Figure S1. YadA and YFC are outer membrane proteins in *Ft*. *Ft*:- *Ft*:YadA, and *Ft*:YFC whole cell (WC) lysates were partitioned into aqueous (A), detergent (D), sarkosyl soluble (SS), and sarkosyl insoluble (SI) phases which were resolved via SDS-PAGE and probed by western blot. (**a**) Primary: rabbit α-YadA detected with goat α-rabbit HRP. (**b**) Top, primary: goat α-C3d detected with biotinylated donkey α-goat and SA-HRP. Bottom, primary: mouse α-FLAG detected with biotinylated goat α-mouse and SA-HRP. The ~18 kDa band in the WC and A phases is an endogenously biotinylated bacterial *Ft* protein, AccB, detected by the streptavidin-HRP conjugate.

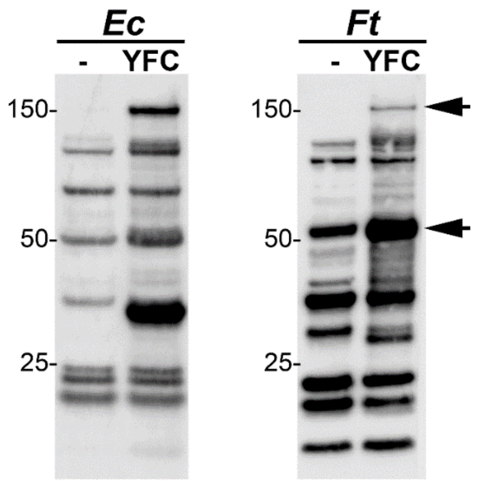

Figure S2. Detection of YFC trimers in *Ec* and *Ft*. Expression of plasmid-borne YFC by *Ec* and *Ft* detected by western blot with α-FLAG, followed by biotinylated secondary and streptavidin-HRP conjugate. Strains bearing an empty vector are denoted by "-". *Ec* strains were grown at room temperature, *Ft* at 37 ^{o}C. Arrows indicate the 154 kDa trimer and the 51 kDa monomer of YFC.

.

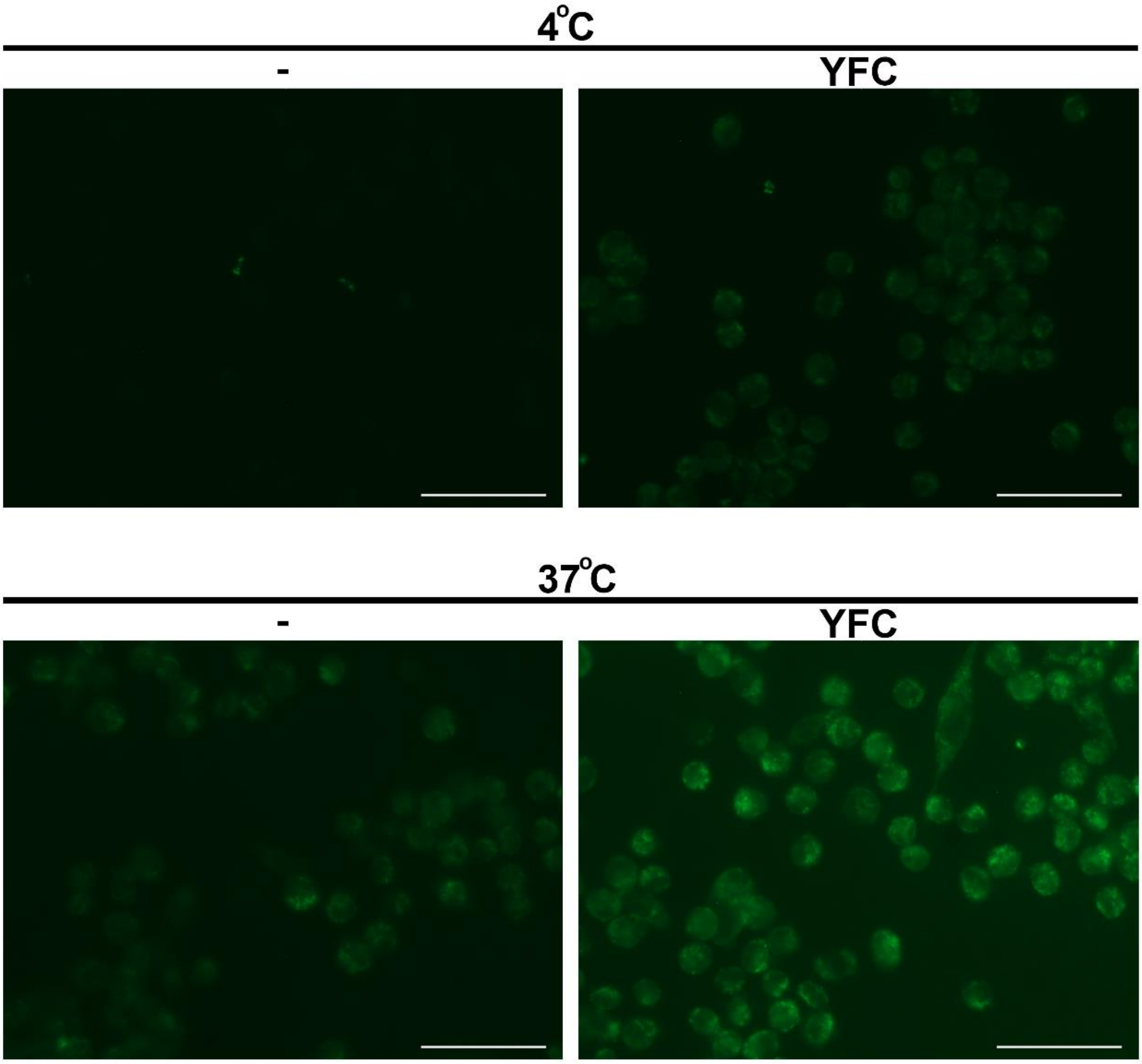

Figure S3. Expanded Figure 2a,b inset images: YFC enhances association of *Ft* to RAW 264.7 cells. SYTO-stained *Ft* strains were incubated with RAW 264.7 cells at an MOI of 100. Scale bars (bottom right) are 50 μm.

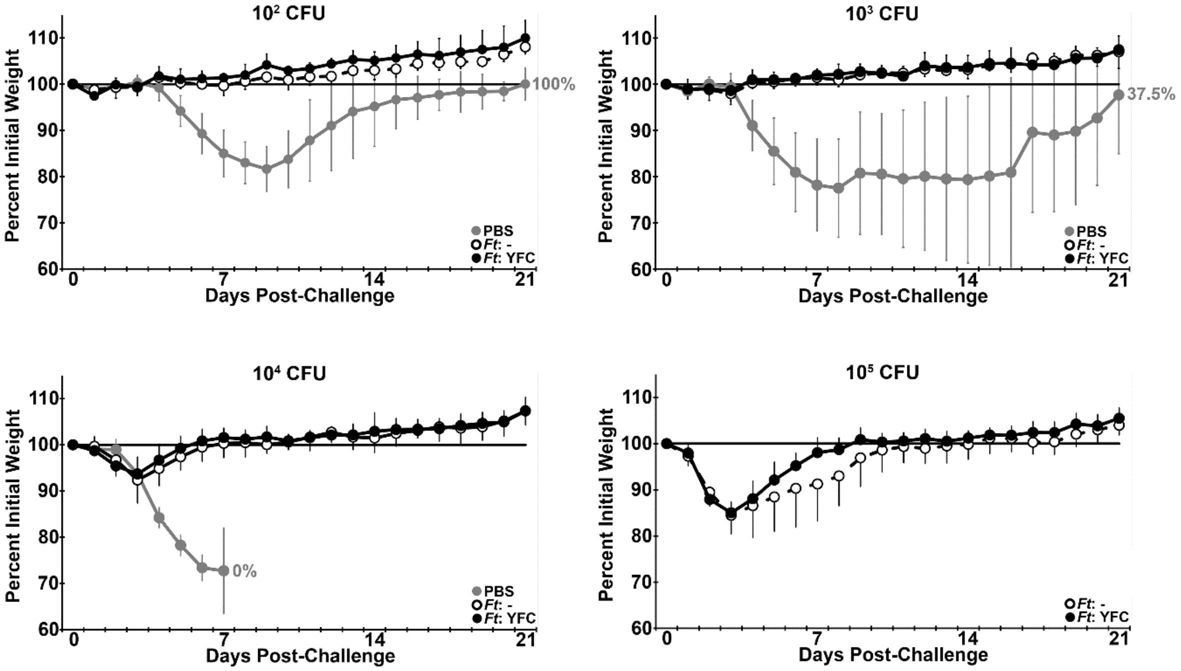

Figure S4. Weight loss following RML challenge. Mice vaccinated with *Ft*:-, *Ft*:YFC, or PBS were challenged on d 28 PV with either 10^2, 10^3, 10^4, or 10^5 CFU of *Ft* LVS RML and their individual weights were recorded daily. All *Ft*:- and *Ft*:YFC vaccinated mice survived all challenge doses. 100% of PBS control mice challenged with 10^2 CFU survived, 37.5% of PBS control mice survived a 10^3 CFU challenge, and all PBS control mice succumbed to the 10^4 CFU challenge. 5-8 mice per group.

References

1. Charity, J.C.; Costante-Hamm, M.M.; Balon, E.L.; Boyd, D.H.; Rubin, E.J.; Dove, S.L. Twin RNA polymerase-associated proteins control virulence gene expression in *Francisella tularensis*. *PLoS. Pathog.* **2007**, *3*, e84.
